# Complete mitochondrial genome of the branching octocoral *Paramuricea grayi* (Johnson, 1861), phylogenetic relationships and divergence analysis

**DOI:** 10.1080/23802359.2022.2143246

**Published:** 2022-11-15

**Authors:** Márcio A. G. Coelho, Jean-Baptiste Ledoux, Joana Boavida, Diogo Paulo, Daniel Gómez-Gras, Nathaniel Bensoussan, Paula López-Sendino, Carlo Cerrano, Silvija Kipson, Tatjana Bakran-Petricioli, Joaquim Garrabou, Ester A. Serrão, Gareth A. Pearson

**Affiliations:** a Centre of Marine Sciences (CCMAR), University of Algarve, Faro, Portugal; b MARE - Marine and Environmental Sciences Centre, ISPA-Instituto Universitário, Lisboa, Portugal; c CIIMAR/CIMAR, Centro Interdisciplinar de Investigação Marinha e Ambiental, Universidade do Porto, Porto, Portugal; d Aix Marseille University, Université de Toulon, CNRS, IRD, MIO UM 110, Marseille, France; e Departament de Biologia Marina, Institut de Ciències del Mar (CSIC), Barcelona, Spain; f Departament de Biologia Evolutiva, Ecologia i Ciències Ambientals, Universitat de Barcelona (UB), Barcelona, Spain; g Institut de Recerca de la Biodiversitat (IRBio), Universitat de Barcelona (UB), Barcelona, Spain; h Dipartimento di Scienze della Vita e dell’Ambiente (DiSVA), Università Politecnica delle Marche, Ancona, Italy; i Consorzio Nazionale Interuniversitario per le Scienze del Mare (CoNISMa), Rome, Italy; j Stazione Zoologica Anton Dohrn, Naples, Italy; k Fano Marine Center, Fano, Italy; l Department of Biology, Faculty of Science, University of Zagreb, Zagreb, Croatia; m SEAFAN – Marine Research & Consultancy, Zagreb, Croatia; n CIBIO/InBIO-Centro de Investigação em Biodiversidade e Recursos Genéticos, Vairão, Portugal

**Keywords:** Octocorallia, gorgonian, Gray’s sea fan, mitogenome, RNA-seq

## Abstract

The Gray’s sea fan, *Paramuricea grayi* (Johnson, 1861), typically inhabits deep littoral and circalittoral habitats of the eastern temperate and tropical Atlantic Ocean. Along the Iberian Peninsula, where *P. grayi* is a dominant constituent of circalittoral coral gardens, two segregating lineages (yellow and purple morphotypes) were recently identified using single-copy nuclear orthologues. The mitochondrial genomes of 9 *P. grayi* individuals covering both color morphotypes were assembled from RNA-seq data, using samples collected at three sites in southern (Sagres and Tavira) and western (Cape Espichel) Portugal. The complete circular mitogenome is 18,668 bp in length, has an A + T-rich base composition (62.5%) and contains the 17 genes typically found in Octocorallia: 14 protein-coding genes (*atp6*, *atp8*, *cob*, *cox1-3*, *mt-mutS*, *nad1-6,* and *nad4L*), the small and large subunit rRNAs (*rns* and *rnl*), and one transfer RNA (*trnM*). The mitogenomes were nearly identical for all specimens, though we identified a noteworthy polymorphism (two SNPs 9 bp apart) in the *mt-mutS* of one purple individual that is shared with the sister species *P. clavata*. The mitogenomes of the two species have a pairwise sequence identity of 99.0%, with *nad6* and *mt-mutS* having the highest rates of non-synonymous substitutions.

*Paramuricea grayi* (Johnson, 1861), colloquially referred to as Gray’s sea fan, is an octocoral species typically inhabiting deep littoral and circalittoral habitats (20-200 m depth; with occasional reports from the upper bathyal zone) of the eastern temperate and tropical Atlantic Ocean, from the Lusitanian-Mauritanian East Atlantic south to the Gulf of Guinea and Angola (Grasshoff 1977, 1992; Altuna 1991). Although historically described as an eastern Atlantic species, recent studies have extended the distribution range of *P. grayi* to the NW Atlantic (Walting et al. 2011; Thoma 2013) and Mediterranean Sea (Quattrini et al. 2022). In regions like the Algarve in southern Portugal, *P. grayi* is an important constituent of circalittoral coral gardens, often dominating coral communities and occurring at high densities despite potentially severe impacts from bottom-contact fisheries (Dias et al. 2020; Nestorowicz et al. 2021).

We recently determined the identity of multiple populations of *Paramuricea* in southern and western Portugal previously identified as *P. clavata* to be *P. grayi*, and identified two color morphotypes of *P. grayi* as segregating lineages based on multi-locus genotyping and phylogenomic analyses (Coelho et al. 2022; Figure 1). While *P. clavata* and *P. grayi* can be distinguished using the *mt-mutS* barcode, the two lineages of *P. grayi* share the same haplotype for this barcode (Coelho et al. 2022). This highlights the need to further study genetic differentiation between *P. clavata* and *P. grayi*, as well as between the two segregating lineages of *P. grayi* at other mitochondrial genes. Here, we report the complete mitochondrial genome (mitogenome) of *P. grayi* assembled from RNA-seq read data generated in Coelho et al. (2022). In total, we assembled complete/partial mitogenomes of nine individuals of *P. grayi*, including specimens of both the yellow (*n* = 7) and purple (*n* = 2) morphotypes. The samples were collected at three sites in southern (Sagres and Tavira) and western (Cape Espichel) Portugal. A specimen of each color morphotype are deposited at the Biogeographical Ecology and Evolution team collection at the Centre of Marine Sciences (email: macoelho@ualg.pt) under voucher IDs 19-0054 and 19-0046. The mitogenomes of *P. clavata* collected in the Mediterranean (Spain, Italy and Croatia; Coelho et al. 2022) and of *P. biscaya* from the Gulf of Mexico (see DeLeo et al. 2018) were also assembled for phylogenetic analysis (see below). For additional information about the origin and collection of samples, as well as about RNA extraction and sequencing see Gómez-Gras et al. (in review), Coelho et al. (2022) and Supplemental Materials Section S1. Quality-filtered, ribosomal RNA-free read data for the samples from Coelho et al. (2022) used here are deposited on NCBI's Sequence Read Archive (BioProject ID: PRJNA847883).

**Figure 1.**
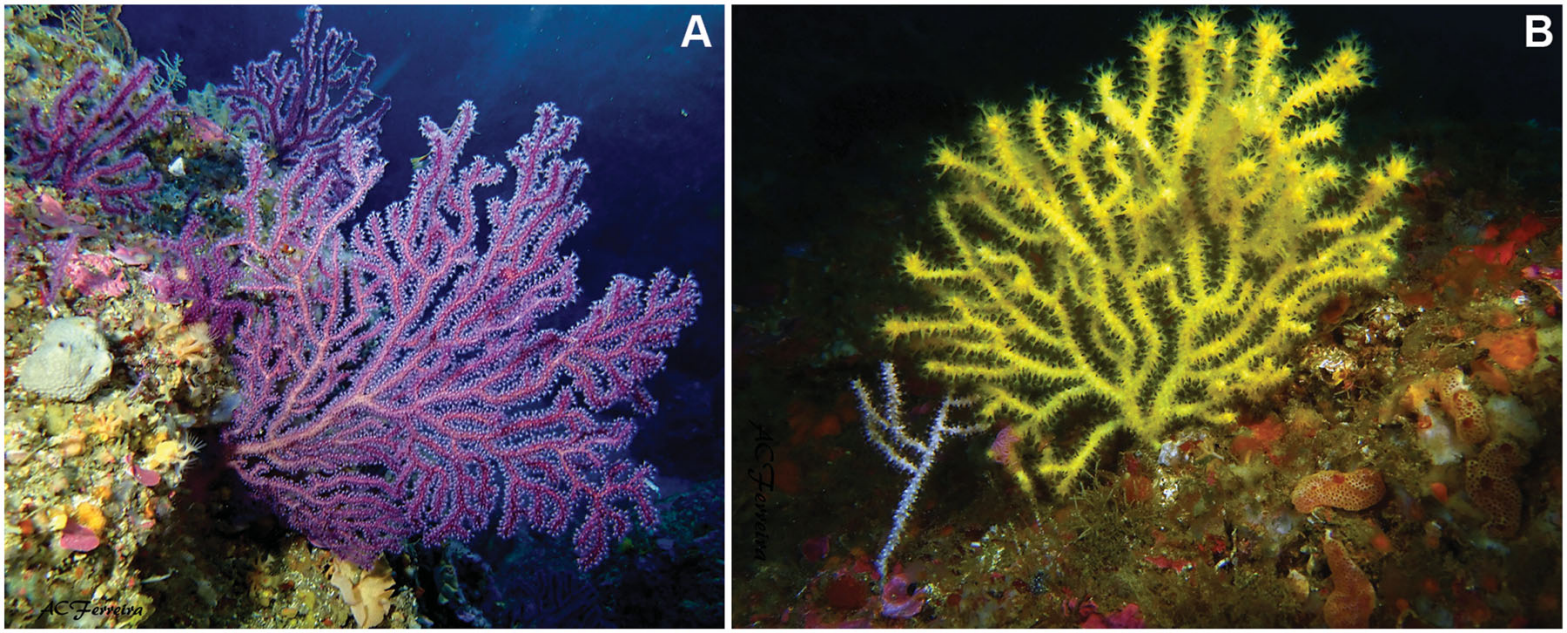
Colonies of *Paramuricea grayi* belonging to the purple (A) and yellow (B) morphotypes corresponding to the segregating lineages identified in Coelho et al. (2022). Colonies were photographed at two distinct sites off Cape Espichel in western Portugal. The colonies of both color morphs tend to have relatively thin branches compared to the Mediterranean sister species *P. clavata* (though colonies with thick branches can also be observed in *P. grayi*, especially in the purple morphotype) and change color when dried or preserved (e.g. in etOH 96%), turning into a black/dark brown coloration. Photo credits: A.C. Ferreira.

The mitogenomes were assembled with MITGARD (**Mit**ochondrial **G**enome **A**ssembly from **R**NA-seq **D**ata) using default parameters (Nachtigall et al. 2021). MITGARD is an automated pipeline that allows mitochondrial sequence reads to be retrieved from RNA-seq data using a reference mitogenome as bait to subsequently generate *de novo* contigs and a consensus mitogenome assembly. We used the mitogenome sequence of *P. clavata* as a reference (Genbank Accession: NC_034749). The assembled mitogenomes were then visually curated by examining the alignment files of mapped reads, as well as of the assembled contigs and consensus mitogenome, onto the *P. clavata* reference in Geneious Prime v2022.0.1. This analysis revealed sequence incongruencies for some specimens in regions with consistently low read coverage across samples (e.g. regions spanning the 3'-end of *cox1* and *rnl*; see Supplemental Materials, Section S2). For this reason and due to widespread (virtual) lack of intra-population polymorphism at mitochondrial genes in Octocorallia (e.g. Calderón et al. 2006; Hooper et al. 2021), the assembled mitogenomes of specimens with the highest read coverage were validated by comparison against the population consensus sequences (*P. clavata*: VAC, ALT and BALU; *P. grayi*: BAL; see Section S1 for details on population codes), as well as against a mitogenome assembled with long-read sequencing data for *P. grayi* (Costa et al. unpublished data). The assembled mitogenomes of both *P. grayi* and *P. clavata* were annotated using MITOS2 (Donath et al. 2019). Genes that were not identified (only *rns*), as well as gene boundaries in disagreement with available references (e.g. *cox1* and *rnl*) were manually added in Geneious Prime. Finally, we performed a maximum likelihood (ML) phylogenetic analysis in IQ-TREE 2 (Minh et al. 2020) based on the mitogenomes of multiple octocorals (for details see Supplemental Materials, Section S3); and calculated the rate of synonymous (*d*S) and non-synonymous (*d*N) substitutions for protein-coding genes between *P. grayi* and *P. clavata* (NC_034749) using the online platform PAL2NAL (Suyama et al. 2006).

The complete circular mitogenome of *P. grayi* is 18,668 bp in length, has an A + T-rich base composition (62.5%) and contains the 17 genes typically found in Octocorallia: 14 protein-coding genes, including 13 energy pathway proteins (*atp6*, *atp8*, *cob*, *cox1-3*, *nad1-6,* and *nad4L*) and the mitochondrial homologue of the DNA mismatch repair MutS-like protein (*mt-mutS*); the small and large subunit rRNAs, *rns* and *rnl*, respectively; and one transfer RNA (*trnM*; methionine) (Figure 2). Like other members of family Paramuriceidae (formerly Plexauridae; see McFadden et al. 2022), *P. grayi* has the presumed ancestral octocoral gene order A (Brockman and McFadden 2012), with 12 genes encoded in the heavy strand (*cox1-rns-nad1-cob-nad6-nad3-nad4L-mt-mutS-rnl-nad2-nad5-nad4*) and 5 genes in the light strand (*trnM-cox3-atp6-atp8-cox2*). In total, the intergenic regions (IGR) accounted for 618 bp of the mitogenome, ranging between 5 bp (*rns-nad1* and *rnl-nad2* IGRs) and 112 bp (*cox2-cox1* IGR). The gene boundaries of *nad2* and *nad5* overlapped by 12 bp (Figure 2; Supplemental Materials, Section S4). The partial/complete mitogenome sequences of *P. grayi* were nearly identical for all nine individuals, including across the two segregating color morphotypes. Notable exceptions included low-confidence regions with low/no read coverage (Section S2); one single nucleotide polymorphism (SNP) in *nad6* for one of the seven individuals of the yellow lineage (BAL_8; 439 X coverage); and two SNPs 9 bp apart in *mt-mutS* for one of the two purple individuals (59X and 56X coverage), a polymorphism shared with *P. clavata*.

**Figure 2.**
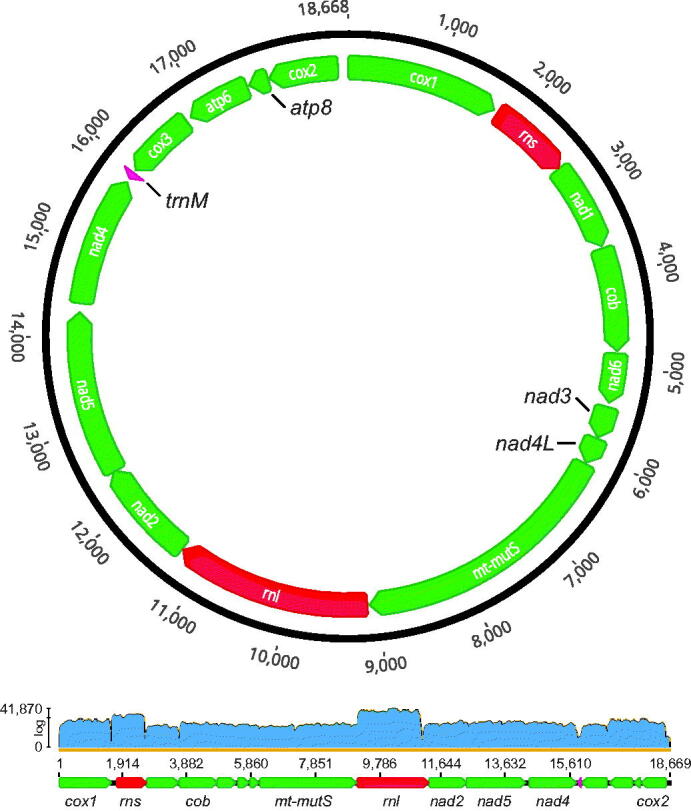
Circular map of the complete mitochondrial genome (i.e. mitogenome) of *Paramuricea grayi* (sample BAL_3; Sequence Read Archive (SRA) and GenBank accessions: SRR19977454 and SRR19977458, and OP205061, respectively). Note that there are two SRA accession numbers that correspond to RNA-seq read data from the same specimen sampled at two distinct time points during a heat-stress experiment. Arrows indicate the direction of transcription, with 12 genes encoded in the heavy strand (*cox1-rns-nad1-cob-nad6-nad3-nad4L-mt-mutS-rnl-nad2-nad5-nad4*) and 5 genes in the light strand (*trnM-cox3-atp6-atp8-cox2*). The genome is 18,668 bp in length and has the presumed ancestral octocoral gene order A (Brockman and McFadden 2012). The lower panel shows read coverage along the linearized reference mitogenome of *P. clavata* (Genbank Accession: NC_034749) obtained with MITGARD for the mitogenome assembly of sample BAL_3. Note that only a subset of the genes are labeled in the linearized graph. The average coverage was 3089X (range: 3–41,870X). For additional information about gene boundaries see Supplemental Materials, Section S4.

Like previous studies, our ML analysis based on mitogenome sequence data show that *P. grayi* and *P. clavata* are recently diverged sister species (Poliseno et al. 2017; Coelho et al. 2022; Quattrini et al. 2022; Figure 3). The mitogenomes of the two species have a pairwise sequence identity of 99.0%, with 87.2% of those mutations in protein-coding genes. The *d*S, a proxy for the mutation rate, ranged between 0.0000 (*atp8*) and 0.0602 (*cox3*), with the *cox1* and *mt-mutS* genes, which encompass the target regions commonly employed for animal and octocoral barcoding, respectively, showing similar *d*S values (0.0385 vs. 0.0425, respectively; Supplemental Materials, Section S5). In contrast, none of the mutations observed in *cox1* resulted in amino acid replacements, whereas *mt-mutS* had 12 non-synonymous mutations (*d*N = 0.0058). Surprisingly, for the *P. grayi*-*P. clavata* comparison *nad6* had the highest *d*N of all protein-coding genes (0.0071; Section S4), and together with *cox2* the highest *d*N/*d*S ratios (Section S4; for a comprehensive overview of the evolution of mitochondrial protein-coding genes in octocorals see Muthye et al. 2022). With the rapid expansion of next- and third-generation sequencing projects, data from RNA-seq represent an underused, but valuable resource to assemble octocoral mitochondrial genomes and more broadly to study the evolution of the eukaryotic powerhouse.

**Figure 3.**
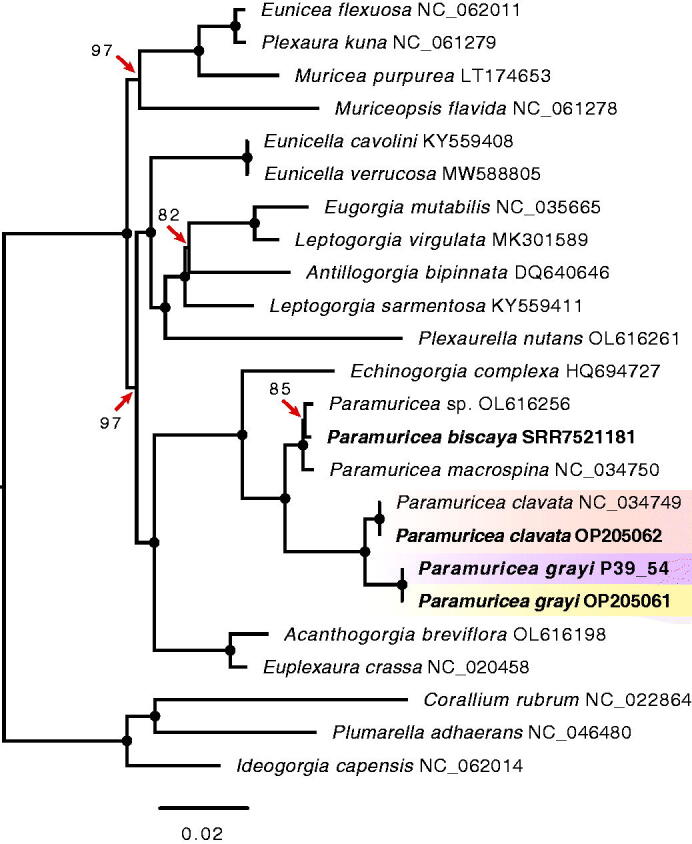
Phylogenetic relationships in 24 octocorals determined by maximum likelihood partition analysis in IQ-TREE 2. The analysis included species from seven families within order Alcyonacea (soft corals and gorgonians). The tree was built using the alignments of 14 mitochondrial protein-coding genes with a separate evolutionary model per gene (for details see Supplemental Materials, Section S3). Branches with full ultrafast bootstrap support (1000 replicates) are indicated by circles, with lower values shown on the branches. Mitogenomes assembled here are shown in bold, with gradient boxes highlighting the specimens of the purple and yellow morphotypes of *Paramuricea grayi* and the sister species *Paramuricea clavata*. Note that the mitogenome sequence of the purple individual P39_54 differed from the remaining eight *P. grayi* mitogenomes assembled (including another individual of the purple morphotype) by two polymorphisms located in the *mt-mutS* gene (9 bp apart), which are also found in *P. clavata*. Close inspection of read data in other specimens revealed multiple reads containing these two SNPs in the other purple individual (P39_57) despite of the consensus sequence matching the SNP pattern found in the yellow lineage of *P. grayi*. Note that the two SNPs identified in the *mt-mutS* are located downstream of the region of this gene commonly used to barcode octocorals. Compared to the reference available on Genbank, the *P. clavata* mitogenomes assembled here differed from the reference in four polymorphisms, one SNP in the *mt-mutS*, as well as a 1 bp deletion in the *rnl* and two SNPs in *nad6* also observed in all of the *P. grayi* mitogenomes. Accession numbers of sequences retrieved from NCBI GenBank or Sequence Read Archive (SRA) are embedded in the taxon names, including *P. biscaya* for which the mitogenome was assembled here using RNA-seq data generated previously by DeLeo et al. (2018). GenBank accession numbers for the complete mitogenome sequences of *P. clavata* (sample VAC_1) and *P. grayi* (sample BAL_3) are OP205061 and OP205062, respectively. The partial/complete sequences of the remaining mitogenome assemblies, including the purple individual P39_54 used in the phylogeny are deposited in Figshare (DOI: 10.6084/m9.figshare.20526978). For additional information about SRA accessions of raw read data for each sample, see Supplemental Materials Section S1.

## Supplementary Material

Supplemental Material

## Data Availability

Raw RNA-seq read data are available at NCBI's SRA database (BioProject ID: PRJNA847883). The complete assembled mitogenomes of *P. grayi* (specimen BAL_3; Biosample [SRA] accessions: SAMN28899305 [SRR19977458] and SAMN28899308 [SRR19977454]) and P*. clavata* (specimen VAC_1; Biosample accessions: SAMN28899326 [SRR19977435] and SAMN28899323 [SRR19977438]) have been submitted to GenBank under accession numbers OP205061 and OP205062, respectively. Partial/complete mitogenome sequences of all the assemblies performed here (including *P. biscaya*) are available on Figshare (DOI: 10.6084/m9.figshare.20526978).
