# Supplementary material for: Complete mitochondrial genome of the branching octocoral Paramuricea grayi (Johnson, 1861), phylogenetic relationships and divergence analysis
Source: Mitochondrial DNA B Resour. 2022 Nov 15;7(11):1985–8. doi: 10.1080/23802359.2022.2143246 (PMC9673786; doi:10.1080/23802359.2022.2143246)
Supplement: Supplemental Material [file TMDN_A_2143246_SM1284.docx]

**Supplemental Materials for:**

**Complete mitochondrial genome of the branching octocoral *Paramuricea grayi* (Johnson, 1861), phylogenetic relationships and divergence analysis**

Márcio A. G. Coelho^1,2*^, Jean-Baptiste Ledoux^3^, Joana Boavida^4^, Diogo Paulo^1^, Daniel Gómez-Gras^5,6,7^, Nathaniel Bensoussan^4,5^, Paula López-Sendino^5^, Carlo Cerrano^8,9,10,11^, Silvija Kipson^12,13^, Tatjana Bakran-Petricioli^12^, Joaquim Garrabou^4,5^, Ester A. Serrão^1,14^, Gareth A. Pearson^1^

Section S1 – Table summarizing the location, coordinates, depth and sample size from Coelho et al. (2022) used to assemble mitochondrial genomes. For *P.* *grayi*, specimens P39_54 and P39_57 were from the purple lineage, with the remaining specimens from the yellow lineage. Entries with two Sequence Read Archive (SRA) accession numbers refer to pooled sequence data from the same specimen sampled at two distinct time points during the heat-stress experiments Coelho et al. (*unpublished data*). Additional details are given in SRA BioProject PRJNA847883. The number of reads mapped onto the reference mitogenome of *P. clavata* are shown. In addition, we assembled the mitogenome of four samples of *P. biscaya* using RNA-seq data from the study of DeLeo et al. (2018) (SRA accessions: SRX4389727, SRX4389728, SRX4389729 and SRX4389730).

| **Species** | **Location name** | **Location code** | **Latitude** | **Longitude** | **Depth (m)** | **Sample ID** | **SRA accession** | **PE reads** |
| --- | --- | --- | --- | --- | --- | --- | --- | --- |
| *P.* *grayi* | P39 (Cape Espichel, West Iberia) | P39 | 38.412 | -9.241 | 31-35 | P39_54 | SRR19977462 | 54,567 |
| *P.* *grayi* | P39 (Cape Espichel, West Iberia) | P39 | 38.412 | -9.241 | 31-35 | P39_57 | SRR19977461 | 53,322 |
| *P.* *grayi* | P39 (Cape Espichel, West Iberia) | P39 | 38.412 | -9.241 | 31-35 | P39_46 | SRR19977460 | 235,411 |
| *P.* *grayi* | Pedra do Barril (Tavira, Southwest Iberia) | TAV | 37.057 | -7.635 | 26.4 | Tav_97 | SRR19977459 | 77,192 |
| *P.* *grayi* | Baleeira (Sagres, Southwest Iberia) | BAL | 37.012 | -8.924 | 15 | BAL_1 | SRR19977449 & SRR19977452 | 279,627 |
| *P.* *grayi* | Baleeira (Sagres, Southwest Iberia) | BAL | 37.012 | -8.924 | 15 | BAL_3 | SRR19977454 & SRR19977458 | 579,700 |
| *P.* *grayi* | Baleeira (Sagres, Southwest Iberia) | BAL | 37.012 | -8.924 | 15 | BAL_5 | SRR19977448 & SRR19977451 | 402,591 |
| *P.* *grayi* | Baleeira (Sagres, Southwest Iberia) | BAL | 37.012 | -8.924 | 15 | BAL_6 | SRR19977447 & SRR19977450 | 147,637 |
| *P.* *grayi* | Baleeira (Sagres, Southwest Iberia) | BAL | 37.012 | -8.924 | 15 | BAL_8 | SRR19977453 & SRR19977456 | 684,113 |
| *P. clavata* | Balun (Kornati, Croatia) | BALU | 43.805 | 15.255 | 33 - 36 | BALU_1 | SRR19977425 & SRR19977428 | 184,178 |
| *P. clavata* | Balun (Kornati, Croatia) | BALU | 43.805 | 15.255 | 33 - 36 | BALU_3 | SRR19977455 & SRR19977478 | 232,261 |
| *P. clavata* | Balun (Kornati, Croatia) | BALU | 43.805 | 15.255 | 33 - 36 | BALU_4 | SRR19977444 & SRR19977477 | 62,636 |
| *P. clavata* | Balun (Kornati, Croatia) | BALU | 43.805 | 15.255 | 33 - 36 | BALU_6 | SRR19977476 & SRR19977426 | 444,556 |
| *P. clavata* | Balun (Kornati, Croatia) | BALU | 43.805 | 15.255 | 33 - 36 | BALU_7 | SRR19977433 & SRR19977466 | 453,226 |
| *P. clavata* | Altare (Portofino, Italy) | ALT | 44.309 | 9.179 | 35 - 37 | ALT_1 | SRR19977464 & SRR19977467 | 128,212 |
| *P. clavata* | Altare (Portofino, Italy) | ALT | 44.309 | 9.179 | 35 - 37 | ALT_4 | SRR19977470 & SRR19977474 | 282,281 |
| *P. clavata* | Altare (Portofino, Italy) | ALT | 44.309 | 9.179 | 35 - 37 | ALT_6 | SRR19977463 & SRR19977465 | 519,061 |
| *P. clavata* | Altare (Portofino, Italy) | ALT | 44.309 | 9.179 | 35 - 37 | ALT_7 | SRR19977469 & SRR19977473 | 273,510 |
| *P. clavata* | Altare (Portofino, Italy) | ALT | 44.309 | 9.179 | 35 - 37 | ALT_8 | SRR19977468 & SRR19977472 | 561,626 |
| *P. clavata* | La Vaca (Medes, Catalonia, Spain) | VAC | 42.048 | 3.226 | 18 - 20 | VAC_1 | SRR19977435 & SRR19977438 | 553,152 |
| *P. clavata* | La Vaca (Medes, Catalonia, Spain) | VAC | 42.048 | 3.226 | 18 - 20 | VAC_3 | SRR19977441 & SRR19977446 | 237,604 |
| *P. clavata* | La Vaca (Medes, Catalonia, Spain) | VAC | 42.048 | 3.226 | 18 - 20 | VAC_4 | SRR19977440 & SRR19977445 | 571,272 |
| *P. clavata* | La Vaca (Medes, Catalonia, Spain) | VAC | 42.048 | 3.226 | 18 - 20 | VAC_6 | SRR19977434 & SRR19977436 | 278,375 |
| *P. clavata* | La Vaca (Medes, Catalonia, Spain) | VAC | 42.048 | 3.226 | 18 - 20 | VAC_8 | SRR19977442 | 312,757 |

Section S2 – Graphs showing the coverage of mitochondrial sequence reads retrieved with MITGARD against the reference mitogenome of *P. clavata* (Genbank Accession: NC_034749) for each sample. **(a)** *P.* *grayi* with insets (dashed red boxes) showing sequence incongruencies for the contigs assembled with Trinity (Haas et al. 2013) and rnaSPAdes (Bushmanova et al. 2019) (MITGARD dependencies) in areas with typically low read coverage (< 5X). Low-quality regions represented a small fraction of the assembled mitogenomes and tended to occur in regions with consistently low read coverage across samples (e.g., regions spanning the 3′-end of *cox1* **(b)** and *rnl* **(c)** shown for specimen BAL_1). The orange bar (below coverage graphs) in the top panels of **(b)** and **(c)** highlights nucleotide positions in the reference mitogenome with more than 5X coverage. By default, MITGARD assigns an ‘N’ to positions not covered by any assembled contig, which occurred in a few regions for some samples (e.g. 5′-end of *cox1*, *cox2-cox1* IGR and *trnM*). The background colours in **(a)** correspond to individuals of the purple and yellow morphotypes of *P.* *grayi* identified by Coelho et al. (2022). **(d)** and **(e)** read coverage obtained for samples of *P. clavata* from three Mediterranean populations (Balun in Croatia, Altare in Italy and La Vaca in Spain) and *P. biscaya*, respectively. RNA-seq data of *P. biscaya* were sequenced by DeLeo et al. (2018) and retrieved from SRA (see above). The complete mitogenomes of *P. grayi* and *P. clavata* deposited in NCBI Genbank are those of samples BAL_3 and VAC_1. Note that the graphs for coverage are shown on a logarithmic scale and differ between samples.


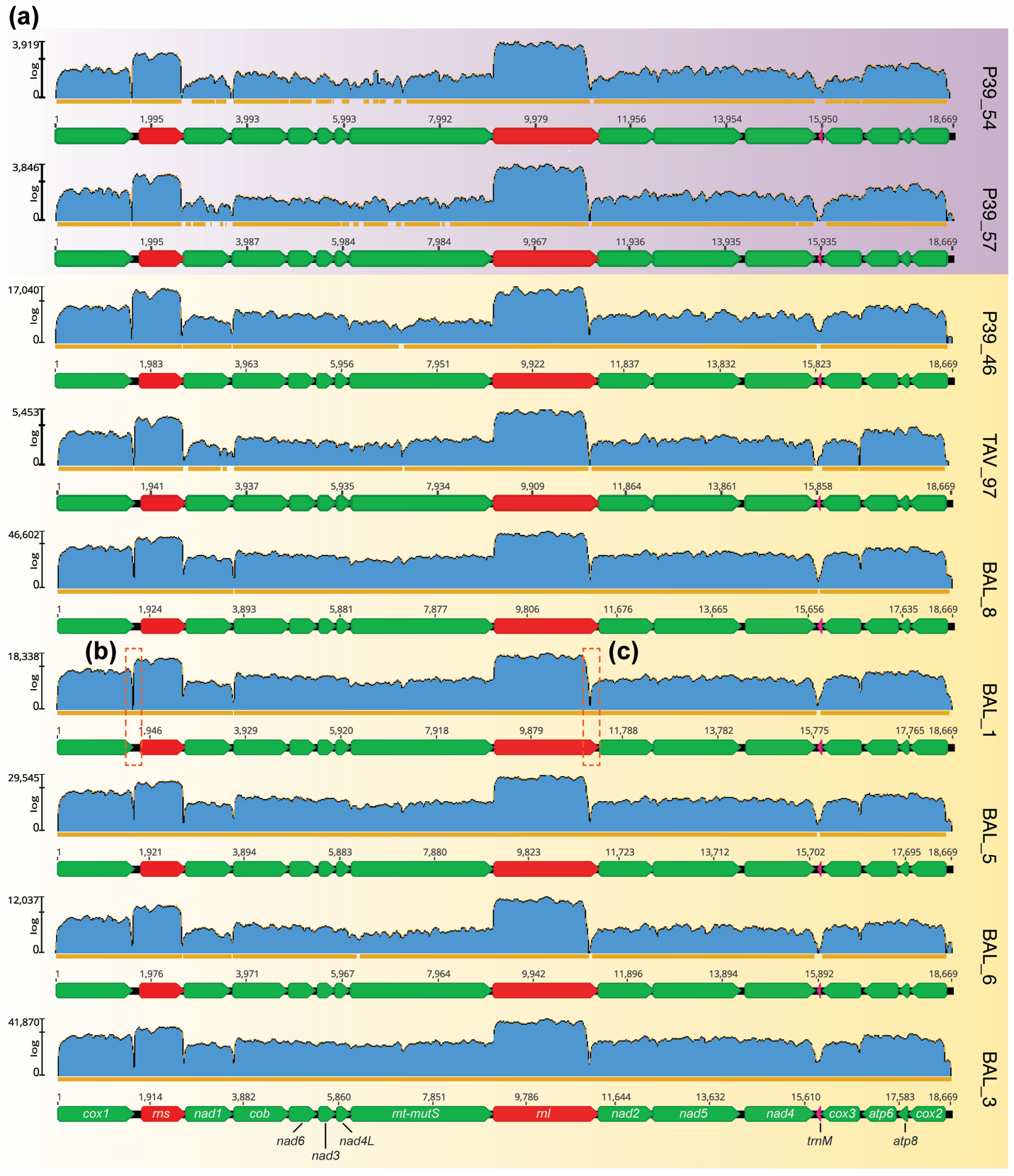


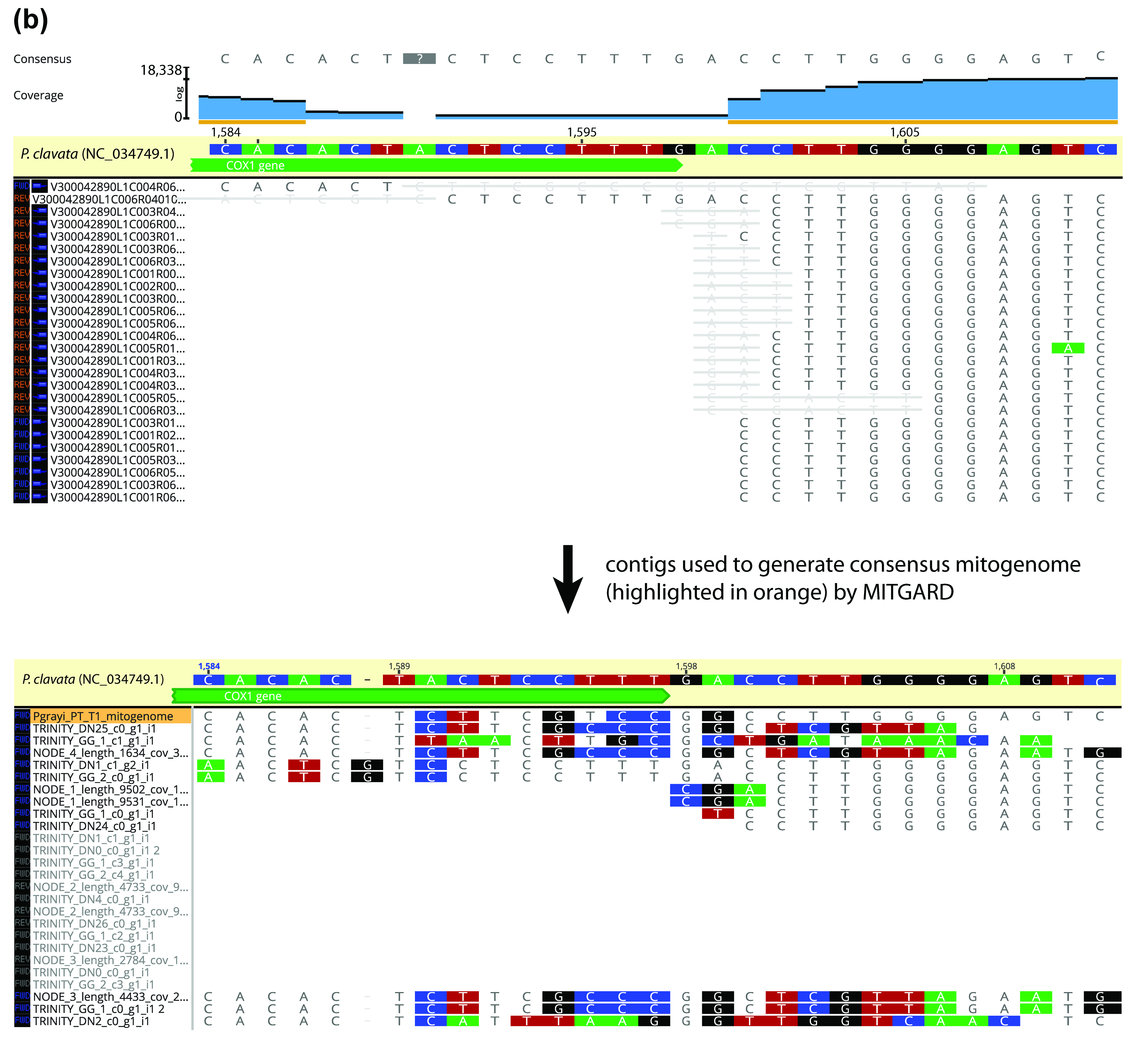


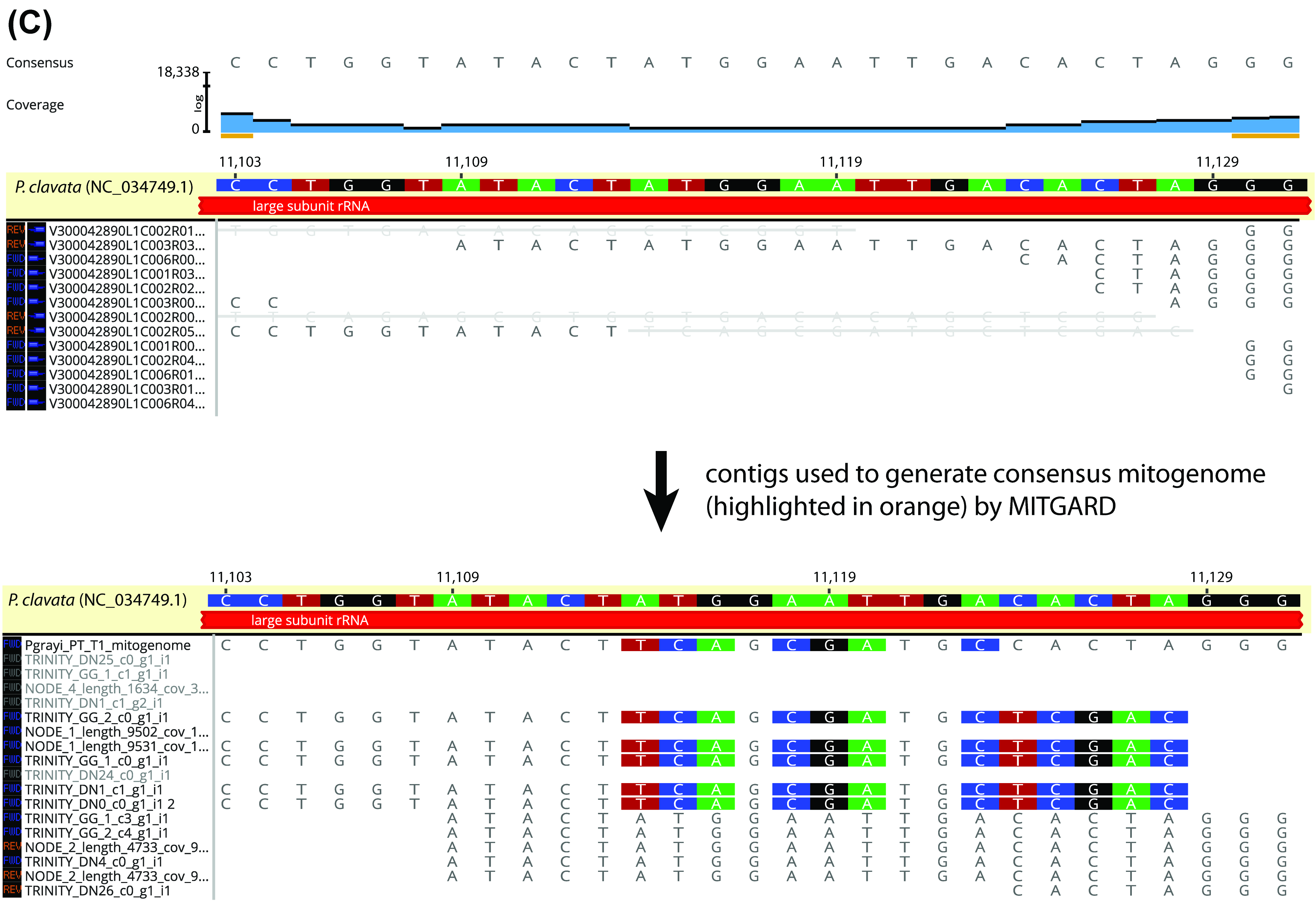


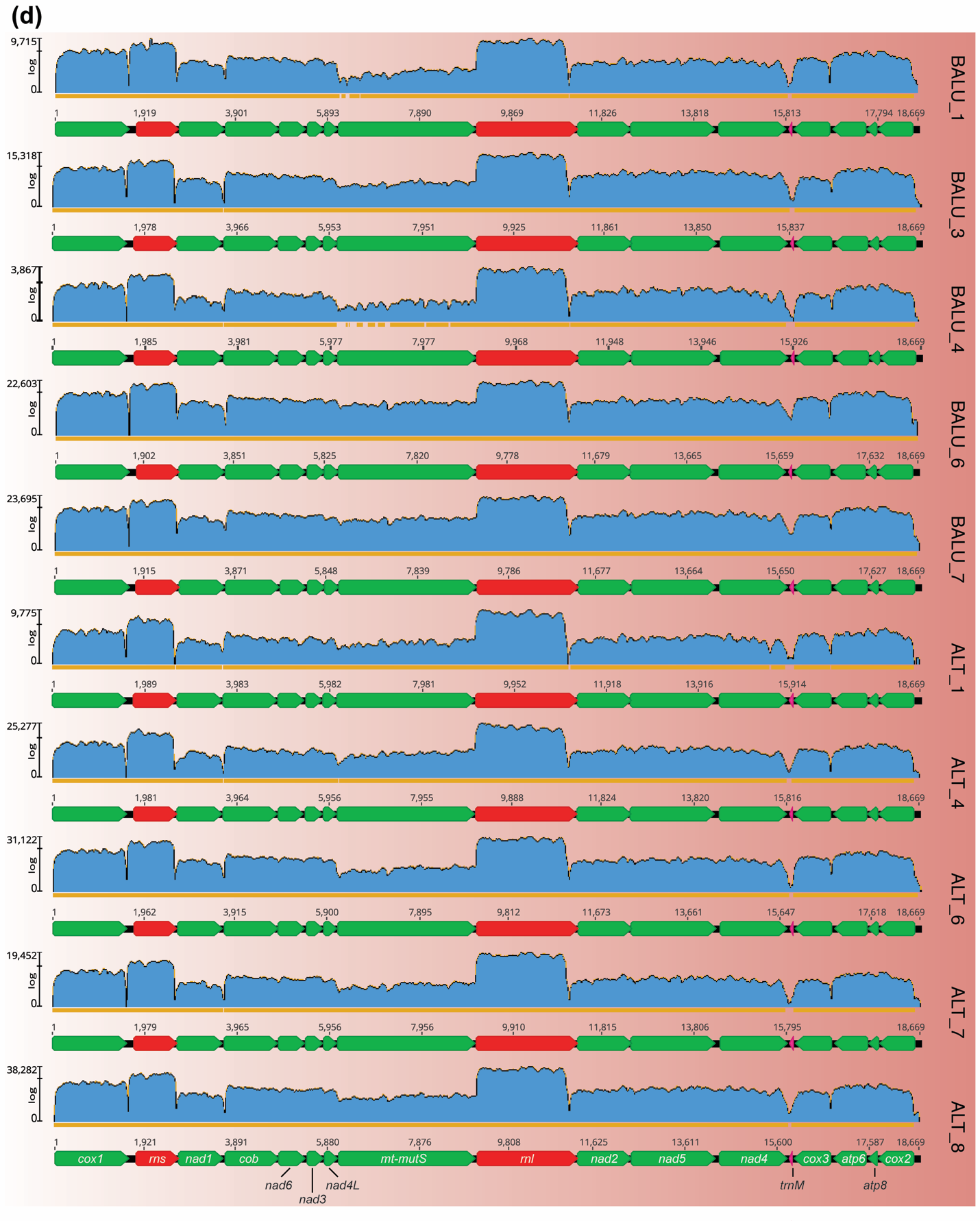


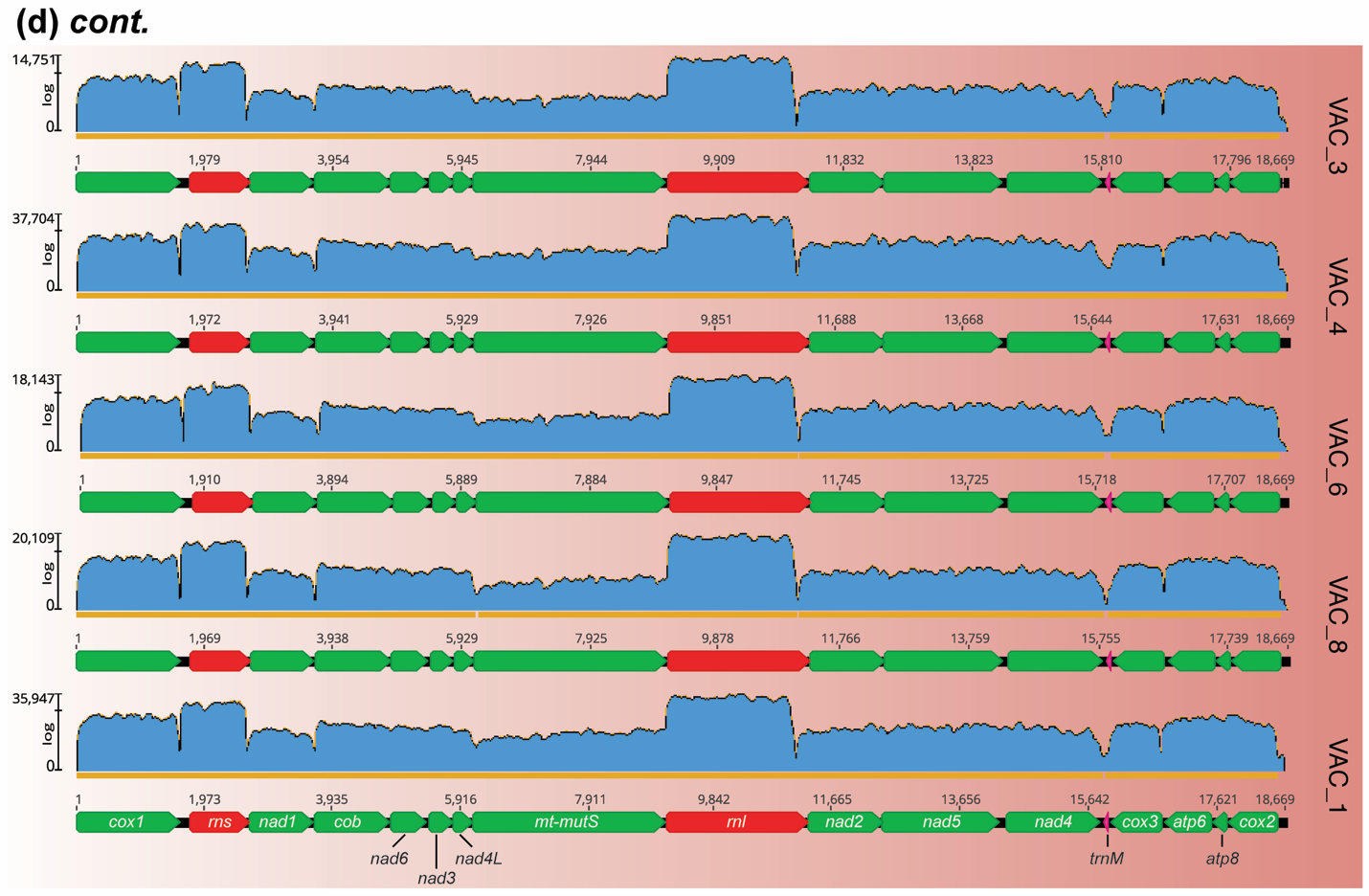


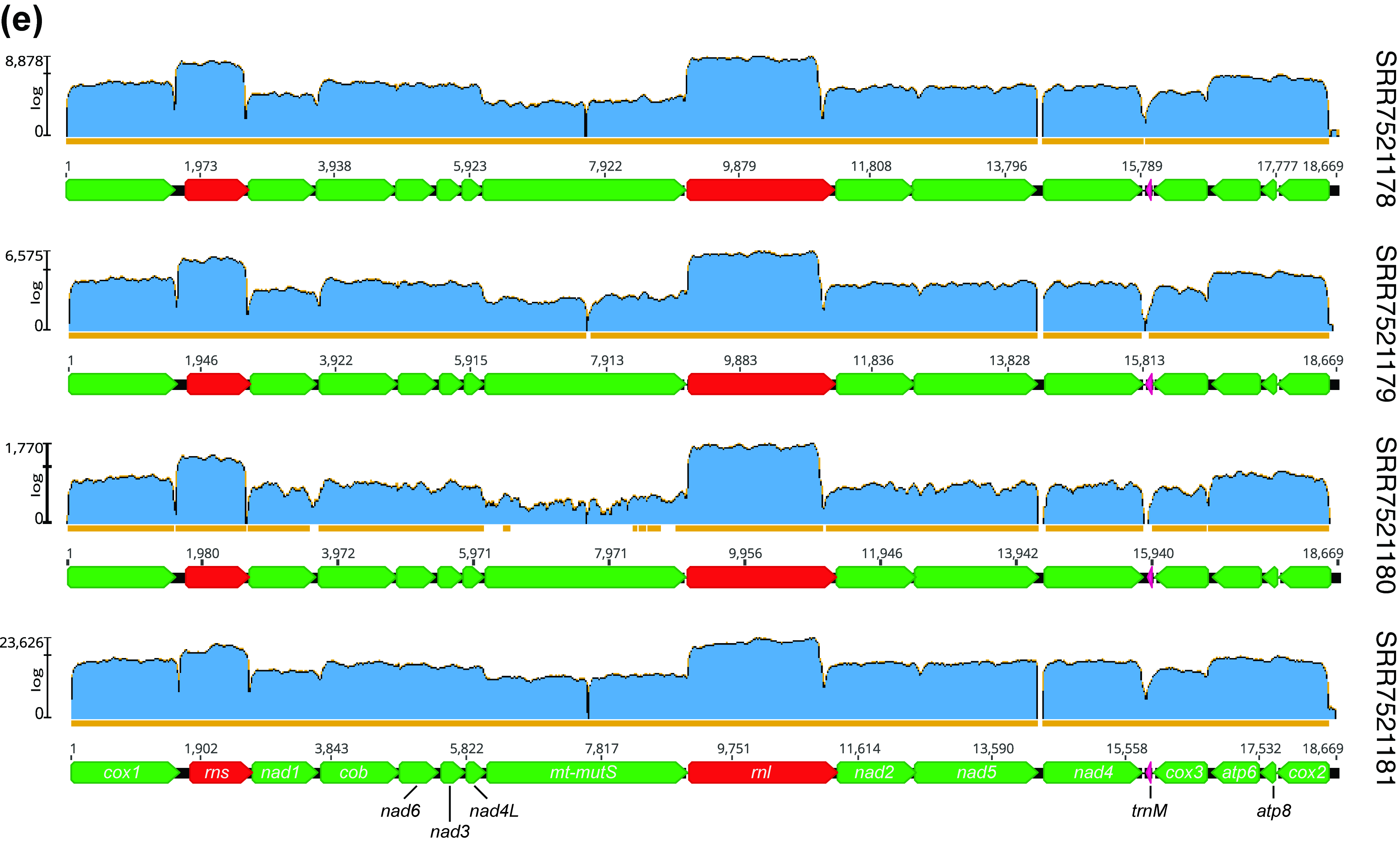


Section S3 –Phylogenetic analysis

The phylogenetic relationships between 24 octocorals were explored by maximum likelihood (ML) in IQ-TREE 2 (Minh et al. 2020a) using 14 protein-coding genes. Sequence data at each locus was aligned separately with the MAFFT plugin in Geneious Prime v2022.0.1 using default parameters for translation alignments (i.e. guided by protein sequences). The Mold Protozoan Mitochondrial genetic code was used for the alignments. The 3′-end of the *mt-mutS* alignment was manually edited to account for a 1 bp deletion in the assembled sequence of *Paramuricea biscaya*. The nucleotide sequences used for *cox1* were based on the longest annotation for the gene that included a stop codon (as opposed to shorter annotations ending in CTTT and which consider the stop codon is completed by the addition of two 3′-adenines to the mRNA strand; Brugler & France 2008; Poliseno et al. 2017). A partitioned ML analysis was performed by fitting a separate evolutionary model of sequence evolution for each locus using ModelFinder (Kalyaanamoorthy et al. 2017) and based on BIC score (Table 3.1). Branch support values were estimated with 1000 ultrafast bootstrap replicates (UFBoot; Hoang et al. 2018).

Table S3.1 - Sequence alignment statistics and models of sequence evolution obtained with ModelFinder for each locus. The full partition model BIC score was 117714.836 (LnL: -58198.325 df:137).

| Gene | # sequences | Sites | Informative | Invariant | Model | Score | Tree Length |
| --- | --- | --- | --- | --- | --- | --- | --- |
| *atp6* | 24 | 708 | 97 | 529 | HKY+F+G4 | 5079.452 | 0.423 |
| *atp8* | 24 | 216 | 29 | 169 | HKY+F+G4 | 1394.912 | 0.375 |
| *cob* | 24 | 1176 | 231 | 837 | HKY+F+G4 | 9848.65 | 0.656 |
| *cox1* | 24 | 1716 | 288 | 1326 | HKY+F+I+G4 | 13046.089 | 0.617 |
| *cox2* | 24 | 762 | 101 | 592 | HKY+F+G4 | 5038.625 | 0.392 |
| *cox3* | 24 | 786 | 96 | 641 | HKY+F+G4 | 4863.385 | 0.335 |
| *mt-mutS* | 24 | 3084 | 762 | 1860 | TPM3+F+G4 | 29753.098 | 0.817 |
| *nad1* | 24 | 981 | 114 | 793 | HKY+F+G4 | 6049.355 | 0.335 |
| *nad2* | 24 | 1158 | 179 | 846 | TPM2+F+R2 | 8690.022 | 0.482 |
| *nad3* | 24 | 360 | 42 | 290 | HKY+F+G4 | 2286.748 | 0.348 |
| *nad4* | 24 | 1449 | 244 | 1075 | TPM2+F+I+G4 | 11063.833 | 0.519 |
| *nad4L* | 24 | 294 | 25 | 254 | HKY+F+G4 | 1483.068 | 0.21 |
| *nad5* | 24 | 1824 | 300 | 1348 | TIM3+F+I+G4 | 14235.946 | 0.556 |
| *nad6* | 24 | 576 | 82 | 431 | HKY+F+G4 | 4196.831 | 0.447 |

Section S4 – Gene annotations for the mitogenome of *Paramuricea grayi* (specimen BAL_3) assembled with MITGARD using RNA-seq data. The boundaries of intergenic regions (IGR) are highlighted in grey, with negative lengths denoting IGRs where gene boundaries overlap. The assembled mitogenome of *Paramuricea clavata* (specimen VAC_1) had length and gene boundaries exactly as in *P.* *grayi*.

| Name | Type | Start | End | Length (bp) | Strand |
| --- | --- | --- | --- | --- | --- |
| *cox1* | gene | 1 | 1,704 | 1,704 | heavy |
| *cox1-rns* | IGR | 1,705 | 1,747 | 43 |  |
| *rns* | rRNA | 1,748 | 2,672 | 925 | heavy |
| *rns-nad1* | IGR | 2,673 | 2,676 | 4 |  |
| *nad1* | gene | 2,677 | 3,648 | 972 | heavy |
| *nad1-cob* | IGR | 3,649 | 3,681 | 33 |  |
| *cob* | gene | 3,682 | 4,836 | 1,155 | heavy |
| *cob-nad6* | IGR | 4,837 | 4,849 | 13 |  |
| *nad6* | gene | 4,850 | 5,407 | 558 | heavy |
| *nad6-nad3* | IGR | 5,408 | 5,450 | 43 |  |
| *nad3* | gene | 5,451 | 5,810 | 360 | heavy |
| *nad3-nad4l* | IGR | 5,811 | 5,822 | 12 |  |
| *nad4l* | gene | 5,823 | 6,116 | 294 | heavy |
| *nad4l-mt-mutS* | IGR | 6,117 | 6,129 | 13 |  |
| *mt-mutS* | gene | 6,130 | 9,093 | 2,964 | heavy |
| *mt-mutS-rnl* | IGR | 9,094 | 9,119 | 26 |  |
| *rnl* | rRNA | 9,120 | 11,297 | 2,178 | heavy |
| *rnl-nad2* | IGR | 11,298 | 11,301 | 4 |  |
| *nad2* | gene | 11,302 | 12,459 | 1,158 | heavy |
| *nad2-nad5* | IGR | 12,460 | 12,446 | -13 |  |
| *nad5* | gene | 12,447 | 14,264 | 1,818 | heavy |
| *nad5-nad4* | IGR | 14,265 | 14,363 | 99 |  |
| *nad4* | gene | 14,364 | 15,812 | 1,449 | heavy |
| *nad4-trnM* | IGR | 15,813 | 15,866 | 54 |  |
| *trnM(cat)* | tRNA | 15,867 | 15,937 | 71 | light |
| *trnM-cox3* | IGR | 15,938 | 15,975 | 38 |  |
| *cox3* | gene | 15,976 | 16,761 | 786 | light |
| *cox3-atp6* | IGR | 16,762 | 16,825 | 64 |  |
| *atp6* | gene | 16,826 | 17,533 | 708 | light |
| *atp6-atp8* | IGR | 17,534 | 17,557 | 24 |  |
| *atp8* | gene | 17,558 | 17,773 | 216 | light |
| *atp8-cox2* | IGR | 17,774 | 17,794 | 21 |  |
| *cox2* | gene | 17,795 | 18,556 | 762 | light |
| *cox2-cox1* | IGR | 18,557 | 18,668 | 112 |  |

Section S5 – Evolution of protein-coding genes in *Paramuricea* showing rates of synonymous (*d*S) and non-synonymous (*d*N) substitutions between *P. grayi* and *P. clavata* (NC_034749), as well as the *d*N/*d*S (*ω*), which can indicate changes in selection pressure. *d*S, *d*N and *ω* were calculated using the online platform PAL2NAL (Suyama et al. 2006).

| Gene | *d*S | *d*N | *d*N/*d*S |
| --- | --- | --- | --- |
| *atp6* | 0.0225 | 0.0018 | 0.0796 |
| *atp8* | 0.0000 | 0.0000 | 0.1000 |
| *cox1* | 0.0385 | 0.0000 | 0.0010 |
| *cox2* | 0.0128 | 0.0034 | 0.2670 |
| *cox3* | 0.0602 | 0.0001 | 0.0010 |
| *cob* | 0.0465 | 0.0012 | 0.0262 |
| *Mt-mutS* | 0.0425 | 0.0058 | 0.1365 |
| *nad1* | 0.0314 | 0.0000 | 0.0010 |
| *nad2* | 0.0384 | 0.0048 | 0.1238 |
| *nad3* | 0.0149 | 0.0000 | 0.0010 |
| *nad4* | 0.0506 | 0.0055 | 0.1095 |
| *nad4L* | 0.0303 | 0.0000 | 0.0010 |
| *nad5* | 0.0592 | 0.0030 | 0.0500 |
| *nad6* | 0.0267 | 0.0071 | 0.2677 |

**References**

Brugler MR, France SC .2008. The Mitochondrial Genome of a Deep-Sea Bamboo Coral (Cnidaria, Anthozoa, Octocorallia, Isididae): Genome Structure and Putative Origins of Replication Are Not Conserved Among Octocorals. J Mol Evol. 67:125–136.

Bushmanova E, Antipov D, Lapidus A, Prjibelski AD. 2019. rnaSPAdes: a de novo transcriptome assembler and its application to RNA-Seq data. GigaSci. 8:1–13.

Coelho MAG, Pearson G, Boavida J, Paulo D, Aurelle D, Arnaud-Haond S, Gómez-Gras D, Bensoussan N, López-Sendino P, Cerrano C, Kipson S, Bakran-Petricioli T, Ferretti E, Linares C, Garrabou J, Serrão E, Ledoux J-B. 2022. Not out of the Mediterranean: Atlantic populations of the gorgonian *Paramuricea clavata* are a separate sister species under further lineage diversification. Authorea. doi: 10.22541/au.165544523.32209087/v1.

DeLeo DM, Herrera S, Lengyel SD, Quattrini AM, Kulathinal RJ, Cordes EE. 2018. Gene expression profiling reveals deep-sea coral response to the Deepwater Horizon oil spill. Mol Ecol. 27:4066–4077.

Haas BJ, Papanicolaou A, Yassour M, Grabherr M, Blood PD, Bowden J, Couger MB, Eccles D, Li B, Lieber M, MacManes MD, Ott M, Orvis J, Pochet N, Strozzi F, Weeks N, Westerman R, William T, Dewey CN, Henschel R, LeDuc RD, Friedman N, Regev A. 2013. *De novo* transcript sequence reconstruction from RNA-seq using the Trinity platform for reference generation and analysis. Nat Protoc. 8:1494–1512.

Hoang DT, Chernomor O, von Haeseler A, Minh BQ, Vinh LS. 2018. UFBoot2: Improving the ultrafast bootstrap approximation. Mol Biol Evol. 35:518–522.

Kalyaanamoorthy S, Minh BQ, Wong TKF, von Haeseler A, Jermiin L. 2017. ModelFinder: Fast Model Selection for Accurate Phylogenetic Estimates. Nat Methods. 14:587–589.

Minh BQ, Schmidt HA, Chernomor O, Schrempf D, Woodhams MD, Haeseler AV, Lanfear R, Teeling E. 2020. IQ-TREE 2: New Models and Efficient Methods for Phylogenetic Inference in the Genomic Era. Mol Biol Evol. 37:1530–1534.

Poliseno A, Altuna A, Cerrano C, Wörheide G, Vargas S. 2017. Historical biogeography and mitogenomics of two endemic Mediterranean gorgonians (Holaxonia, Plexauridae). Org Divers Evol. 17:365–373.

Suyama M, Torrents D, Bork P. 2006. PAL2NAL: robust conversion of protein sequence alignments into the corresponding codon alignments. Nucleic Acids Res 34:W609–W612.
